# Complete mitochondrial genome of a leaf-mining beetle, *Rhadinosa nigrocyanea* (Coleoptera: Chrysomelidae) with phylogenetic consideration

**DOI:** 10.1080/23802359.2017.1357443

**Published:** 2017-07-25

**Authors:** Qingyun Guo, Jiasheng Xu, Xiaohua Dai, Chengqing Liao, Chengpeng Long

**Affiliations:** a Leafminer Group, School of Life and Environmental Sciences, Gannan Normal University, Ganzhou, China;; b National Navel-Orange Engineering Research Center, Ganzhou, China

**Keywords:** Leaf-mining beetle, Coleoptera, mitochondrial genome, *Rhadinosa nigrocyanea*, molecular phylogeny

## Abstract

The complete circular mitochondrial genome (mitogenome) of *Rhadinosa nigrocyanea* was 17,965 bp in length, which contained 2 ribosomal RNA genes, 22 transfer RNAs, 13 protein-coding genes (PCGs) and 1 non-coding AT-rich region with the length of 3002 bp. All of the 22 tRNA genes displayed a typical clover-leaf structure, with the exception of tRNA^Ser^ (TCT). 12 PCGs were initiated by ATN codons, except for ND1 started with TTG. Only six PCGs used the typical stop codon ‘TAA’ and ‘TGA’, while seven PCGs terminated with incomplete stop codons (TA or T). Phylogenetic analysis showed that *R. nigrocyanea* grouped with Cassidinae species, sister to Clytrinae + Cryptocephalinae.

The leaf-mining genus *Rhadinosa* Weise belongs to the tribe Hispini Gyllenhal (Chrysomelidae: Cassidinae), with 12 species occurring mostly in Asia (Staines 2012). The larva of *Rhadinosa nigrocyanea* mines within the leaves of Poaceae such as *Miscanthus floridulus*, producing long strip mines. The genus *Rhadinosa* Weise, 1905 was one of the smallest groups of Cassidinae subfamily and comprised only four species in China (Chen et al. 1986). However, most studies have focused on the morphology, biological characteristics and occurrence pattern of the genus, but its evolutionary history was still limited due to lack of molecular data, to date.

Here, we sequenced and characterized the complete mt genome of *R. nigrocyanea*. The specimen was collected from Anjishan Mountain, Jiangxi Province, China, in June 2015. The adults were kept in the laboratory at −80 °C with 100% ethanol. The total genomic DNA isolated from the head tissue was used to decipher its complete mitochondrial genome. The circular mitogenome of *R. nigrocyanea* was 17,965 bp, A + T rich (76.6%), and includes 37 genes (GenBank accession MF041978) (Table 1), and this size is moderate compared to other Coleoptera species (Bae et al. 2004; Kim et al. 2015). It contains 2 ribosomal RNA genes, 22 transfer RNAs, 13 protein-coding genes (PCGs) and a 3002 bp long non-coding A + T-rich region. Its gene content and orientation are typical of other leaf beetle mitochondrial genomes and identical to those of the putative ancestor of insects (Kim et al. 2015; Song et al. 2017).

**Table 1.** Mitochondrial genome organization for *Rhadinosa nigrocyanea*.

			Size		Codon		
Gene	Strand	Location	(bp)	Inc	Start	Stop	Anticodon
trnI	N	1–65	65	/			GAT
trnQ	J	650–716	67	586			TTG
trnM	N	716–779	64	−1			CAT
nad2	N	780–1754	975	0	ATA	TAA	
trnW	N	1758–1820	63	5			TCA
trnC	J	1813–1873	61	−6			GCA
trnY	J	1874–1934	61	0			GTA
cox1	N	1927–3467	1541	−6	ATT	TA	
trnL2	N	3468–3529	62	0			TAA
cox2	N	3530–4208	679	0	ATT	T	
trnK	N	4209–4278	70	0			TTT
trnD	N	4278–4341	64	−1			GTC
atp8	N	4342–4494	153	0	ATT	TAA	
atp6	N	4488–5150	663	−5	ATG	TAA	
cox3	N	5150–5930	781	−1	ATG	T	
trnG	N	5931–5993	63	0			TCC
nad3	N	5994–6345	352	0	ATT	T	
trnA	N	6346–6407	62	0			TGC
trnR	N	6408–6467	60	0			TCG
trnN	N	6468–6530	63	0			GTT
trnS1	N	6531–6595	65	0			TCT
trnE	N	6596–6657	62	0			TTC
trnF	J	6656–6717	62	−2			GAA
nad5	J	6718–8419	1702	0	ATT	T	
trnH	J	8420–8480	61	0			GTG
nad4	J	8481–9799	1319	0	ATA	TA	
nad4L	J	9801–10080	280	3	ATG	T	
trnT	N	10083–10145	63	4			TGT
trnP	J	10146–10207	62	0			TGG
nad6	N	10209–10685	477	3	ATT	TAA	
cob	N	10685–11824	1140	−1	ATG	TAG	
trnS2	N	11823–11890	68	−2			TGA
nad1	J	11908–12858	951	19	TTG	TAG	
trnL1	J	12860–12922	63	3			TAG
rrnL	J	12952–14176	1225	0			
trnV	J	14177–14238	62	0			TAC
rrnS	J	14237–14963	727	−2			
A + T-rich region	–	14964–17965	3002	/			

N and J refer to the majority and minority strands, respectively.

Inc: intergenic nucleotides; negative values refer to overlapping nucleotides.

The nucleotide composition of the mitogenome of *R. nigrocyanea* is as follows: As (43.25%), Ts (33.36%), Cs (9.51%) and Gs (13.88%), which showed AT bias. 12 PCGs were initiated by ATN codons, except for ND1 started with TTG. In addition, only six PCGs used the typical stop codon ‘TAA’ and ‘TAG’, while seven PCGs terminated with incomplete stop codons (TA or T). All of the 22 tRNAs, ranging from 60 to 70 bp, have a typical clover-leaf secondary structure, except for tRNA^Ser^ (TCT) lacking a stable dihydrouridine stem. The A + T-rich region, AT content of 81.21%, was well known for replication and initiation in both invertebrates and vertebrates (Nardi et al. 2001).

The total length of the 13 PCGs is 10,986 bp with an overall A + T content of 75.1%. The concatenated datasets of the 13 PCGs were used to construct phylogenetic relationships of the eight subfamilies from Chrysomelidae by using maximum parsimony methods (Figure 1). The tree topology was as follows: (((Galerucinae + Chrysomelinae) + Bruchinae) + (Donaciinae + (Cassidinae + (Clytrinae + Cryptocephalinae))) + Criocerinae)))))). The resulting phylogenetic trees strongly supported Cassidinae sister to a clade comprising Clytrinae and Cryptocephalinae (the latter two were sister groups). Thus, *R. nigrocyanea* belonged to Cassidinae species, which was consistent with morphological classification (Staines 2012). These results supported previous research on evolutionary relationships based on mitochondrial genomes in Chrysomelidae (Gómez-Rodríguez et al. 2015).

**Figure 1.**
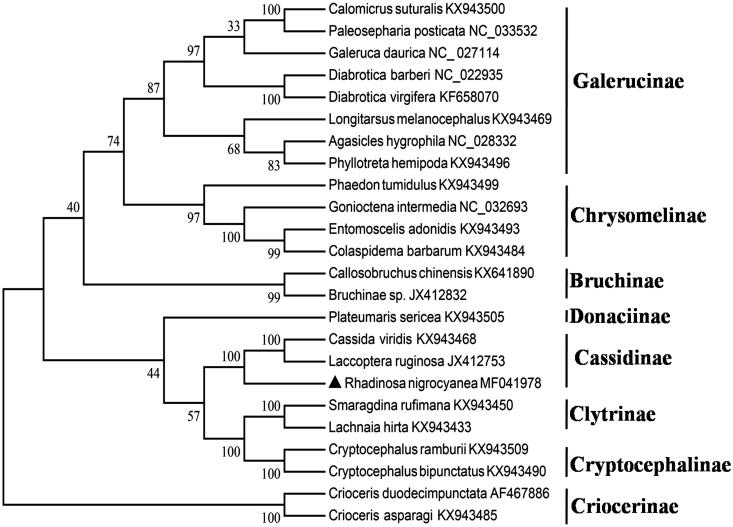
Maximum parsimony tree of *Rhadinosa nigrocyanea* evolutionary relationships based on mitochondrial PCGs catenated dataset.
